# Disturbed natural killer cell homeostasis in the salivary gland enhances autoimmune pathology *via* IFN-γ in a mouse model of primary Sjögren’s syndrome

**DOI:** 10.3389/fmed.2022.1036787

**Published:** 2022-10-26

**Authors:** Mami Sato, Rieko Arakaki, Hiroaki Tawara, Ruka Nagao, Hidetaka Tanaka, Kai Tamura, Yuhki Kawahito, Kunihiro Otsuka, Aya Ushio, Takaaki Tsunematsu, Naozumi Ishimaru

**Affiliations:** Department of Oral Molecular Pathology, Tokushima University Graduate School of Biomedical Sciences, Tokushima, Japan

**Keywords:** NK cell, IFN-γ, T cell, autoimmunity, Sjögren’s syndrome

## Abstract

**Objective:**

Innate lymphoid cells (ILCs), including natural killer (NK) cells, ILC1, ILC2, lymphoid tissue-inducer (LTi) cells, and ILC3 cell, play a key role in various immune responses. Primary Sjögren’s syndrome (pSS) is an autoimmune disease characterized by chronic inflammation of exocrine glands, such as the lacrimal and salivary glands (SGs). The role of NK cells among ILCs in the pathogenesis of pSS is still unclear. In this study, the characteristics and subsets of NK cells in the salivary gland (SG) tissue were analyzed using a murine model of pSS.

**Methods:**

Multiple phenotypes and cytotoxic signature of the SG NK cells in control and pSS model mice were evaluated by flow cytometric analysis. Intracellular expression of interferon-γ (IFN-γ) among T cells and NK cells from the SG tissues was compared by *in vitro* experiments. In addition, pathological analysis was performed using anti-asialo-GM1 (ASGM1) antibody (Ab)-injected pSS model mice.

**Results:**

The number of conventional NK (cNK) cells in the SG of pSS model mice significantly increased compared with that in control mice at 6 weeks of age. The production level of IFN-γ was significantly higher in SG NK cells than in SG T cells. The depletion of NK cells by ASGM1 Ab altered the ratio of tissue resident NK (rNK) cells to cNK cells, which inhibited the injury to SG cells with the recovery of saliva secretion in pSS model mice.

**Conclusion:**

The results indicate that SG cNK cells may enhance the autoreactive response in the target organ by upregulating of IFN-γ, whereas SG rNK cells protect target cells against T cell cytotoxicity. Therefore, the activation process and multiple functions of NK cells in the target organ could be helpful to develop potential markers for determining autoimmune disease activity and target molecules for incurable immune disorders.

## Introduction

Innate lymphoid cells (ILCs), including natural killer (NK) cell, ILC1, ILC2, lymphoid tissue-inducer (LTi) cell, and LIC3, significantly contribute to various immune responses (1–3). ILCs lack adoptive antigen receptors generated by the recombination of genetic elements, unlike T cells, which bear T cell antigen receptors. NK cells and ILC1s react to intracellular pathogens and to tumors in type 1 immunity through interferon-γ (IFN-γ) or cytotoxicity (2, 4, 5). ILC2s respond to large extracellular parasites and allergens in type 2 immunity through interleukin-4 (IL-4), IL-13, or IL-27 (2, 6–8). ILC3s combat extracellular microbes in type 3 immunity *via* IL-22 or IL-17 (2, 9, 10). Additionally, LTi cells are associated with the formation of secondary lymphoid structures (2, 11, 12). Although ILCs contribute to the pathogenesis of infectious or allergic disorders, the differentiation of various immune cells, and the development of lymphoid tissue structures, the precise molecular or cellular mechanisms for the onset or development of autoimmune diseases through ILCs remain unclear.

NK cells, among ILCs, have been focused on when studying the pathogenesis of several systemic or organ-specific autoimmune disorders, such as type 1 diabetes mellitus, primary biliary cholangitis, systemic lupus erythematosus, multiple sclerosis, rheumatoid arthritis, and Sjögren’s syndrome (SS) (13–18). The multiple functions of cytotoxicity and cytokine production with perforin/granzyme and IFN-γ in NK cells are controlled by the expression T-box expressed in T cells (T-bet) *via* the Janus kinase-signal transduction and activation of transcription (JAK-STAT) or phosphatidylinositol-3-kinase-AKT-mammalian target of rapamycine1 (PI3K-AKT-mTORC1) signaling pathway (19, 20). The phenotypes or functions of tissue resident NK cells are considerably different in the target organs of autoimmune diseases (21). Moreover, NK cells are involved in the perpetuation of diseases through the activation of autoreactive T cells in the presence of antigen-presenting cells within the target organ in autoimmunity, such as pSS (18, 22–28). However, the precise relationship between NK cells and autoimmune responses remains unclear.

Primary SS (pSS) is an systemic autoimmune disease characterized by chronic inflammation of exocrine glands, such as SGs, with many different organ-specific manifestations (29–31). Among target organs, the lacrimal glands and SGs are the main target organs in pSS, characterized by progressive lymphocytic infiltration (32). Th1/Th2 cytokine balance or Th17 cells in exocrine glands are involved in the initiation of the autoimmune response in pSS (33–36). IFN-γ-producing Th1 cells play a central role in the pathogenesis of the disease, from the onset to its chronic stage, in addition to various immune cells, such as B cells, macrophages, dendritic cells, and NK cells, in the SGs and lacrimal glands of mouse models of pSS and patients with pSS (18, 37–39). The number of NK cells in the SGs of healthy mice is significantly higher than that of ILC subpopulations (27). In contrast, the detailed cellular or molecular mechanisms related to tissue resident NK cells for the pathogenesis of pSS are unclear.

In this study, the phenotypes and pathogenic role of tissue resident NK cells within the SGs were analyzed using a mouse model of pSS to better understand the autoimmune response between various immune cells, including NK cells in the target organ. Additionally, a new therapy based on the pathogenic role of NK cells was elucidated using the mouse model. The activation process and multiple functions of tissue resident NK cells in the target organ could help develop potential markers for autoimmune disease activity and target molecules for incurable immune disorders.

## Materials and methods

### Mice

Female NFS/N mice carrying the mutant *sld* were bred and maintained in a specific pathogen-free mouse colony in the animal facility at Tokushima University (Tokushima, Japan). Neonatal thymectomy was performed on day 3 after birth to develop pSS model mice. The control mice used in this study were sham (non)-thymectomized NFS/*sld* mice that did not exhibit inflammatory lesions in the SGs and lacrimal glands. This study was conducted according to the Fundamental Guidelines for Proper Conduct of Animal Experiment and Related Activities in Academic Research Institutions under the jurisdiction of the Ministry of Education, Culture, Sports, Science, and Technology of Japan. The study protocol was approved by the Committee on Animal Experiments of Tokushima University, Japan (Permit Number: T-2021-48). All experiments were performed after the administration of anesthesia, and all efforts were made to minimize suffering. Female NFS/*sld* mice (6–12 weeks) were used in this study.

### Administration of anti-asialo GM1 antibody to deplete natural killer cells

Mice were intraperitoneally injected with 100 μg of rabbit anti-asialo GM1 antibody (Fujifilm) or control rabbit IgG twice a week from 9 weeks of age. The mice were euthanized at 12 weeks.

### Cell isolations

Mouse SG suspension cells were prepared for the detection of NK cells using flow cytometry. SG tissues were minced into 1–3 mm^2^ pieces and were digested with 1 mg/mlL collagenase (Fujifilm), 1 mg/mL hyaluronidase (Tokyo Chemical Industry Co., Ltd.) and 10 ng/mL DNase (Roche) in Dulbecco’s Modified Eagle’s Medium (DMEM) containing 10% fetal calf serum at 37°C for 35 min with gentleMACS Dissociators (Miltenyi Biotec). The digested suspension cells were washed and passed through pre-separation filters (20 μm) (Miltenyi Biotec). For the detection of cytokine produced cells, the single cells in the SG were purified using CD45 MicroBeads (Miltenyi Biotec).

### Flow cytometric analysis

Splenocytes and the prepared SG suspension cells were stained using the indicated Abs and 7-amino-actinomycin D (7-AAD) (Biolegend) after pre-incubation with anti-CD16/32 Ab. Abs against mouse fluorescein isothiocyanate (FITC)-conjugated NKp46, phycoerythrin (PE)-conjugated CD49a, CD11b, TRAIL, IFN-γ, DR5, PE-Cyanine7 (Cy7)-conjugated CD19, CD4, allophycocyanin (APC)-conjugated CD49b, CD27, KLRG1, TNF-α, EOMES, APC-Cy7-conjugated CD3, CD8, peridinin chlorophyll protein-cyanine5.5-conjugated CD19, Alexa Fluor^®^ 700 conjugated-CD45.2Abs were purchased from Biolegend. Isotype control Abs for rat IgG1, rat IgG2a, rat IgG2b, rat IgM, and hamster IgG were purchased from Biolegend. A CytoFLEX flow cytometer (Beckman Coulter) was used to identify the cell populations according to expression profile. To determine intracellular cytokine expression, purified CD45.2^+^ cells were stimulated with phorbol myristate acetate (PMA) (50 ng/mL) and ionomycin (1 μg/mL) in the presence of brefeldin A (eBioscience) in a 96-well round bottom plate for 24 h. Cells producing IFN-γ and TNF-α were stained with the indicated Abs using IC Fixation Buffer and Permeabilization Buffer (eBioscience), and were detected using flow cytometry. Viable cells were checked by gating on side scatter (SSC)/forward scatter (FSC), FSC-H/FSC-A, 7AAD. Data were analyzed using the FlowJo FACS Analysis software (Tree Star Inc.).

### Histological analysis

Salivary gland tissues were fixed with 10% phosphate-buffered formaldehyde (pH 7.2) and were prepared for histological examination. Sections were stained with hematoxylin and eosin (HE). Histological changes were histologically graded on a 0–4 scale as described previously (40). The number of lymphocytes that infiltrated into SG tissues per mm^2^ was measured using HE-stained sections (41). The focus number was assessed as a focus with ≥50 mononuclear cells per 2 × 2 mm^2^ in inflammatoy lesions of SG tissues.

### Immunohistochemistry

Tissue sections (5 μm) were deparaffinized in xylenes and were rehydrated by passing through serial dilutions of ethanol in distilled water. Heat-induced antigen retrieval was performed in ImmunoActive antigen retrieval solution (Matsunami Glass Ind. Ltd.) with microwave thrice for 5 min. Goat anti-mouse NKp46 antibody (R&D systems) was applied to the sections; then, the sections were incubated overnight at 4°C. After washing with phosphate-buffered saline (PBS), the sections were incubated with goat IgG horseradish peroxidase (HRP) polymer antibody (R&D systems). HRP reacted with the 3, 3’-diaminobenzidine (DAB) substrate using the SignalStain^®^ DAB Substrate kit (Cell Signaling Technology). The sections were counterstained with hematoxylin.

### Measurement of saliva secretion

All mice were anesthetized with isoflurane (Pfizer) using a small animal inhalation anesthesia machine (Laboratory & Medical Supplies) 5 min before testing and then orally administered with 500 μg/kg body weight of pilocarpine (Fujifilm). The mice were held in a slightly downward tilt, and then, a weighed filter paper was placed into the oral cavity, following which the filter paper was changed every 5 min during the 15 min observation period. The absorbed saliva weight was calculated by subtracting the weight of the filter paper before measurement from that after measurement. The amount of saliva secretion was expressed as the amount of saliva secreted per 10 g of body weight every 5 min.

### Statistical analysis

Differences between individual groups were determined using two-tailed Student’s *t*-test, one-way analysis of variance (ANOVA), or two-way ANOVA. *P*-values of less than 0.05 were used to denote statistical significance. Power calculations were performed before the initiation of the experiments to determine the sample size for experiments using animals. Data are presented as means ± standard errors of the mean.

## Results

### Salivary gland natural killer cells in primary Sjögren’s syndrome model mice

**FIGURE 1:**
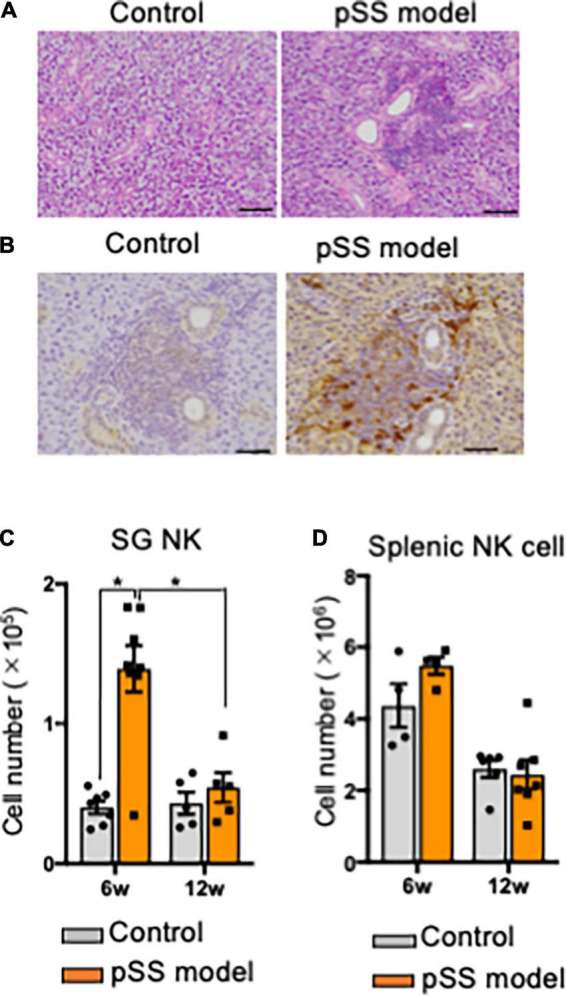
NK cells in salivary glands of pSS model mice. **(A)** Pathological analysis of salivary gland (SG) tissues in control and pSS model mice. Sections stained with hematoxylin and eosin (HE) were used. Photos are representative of each group. Scale bar: 100 μm. **(B)** Immunohistochemical analysis of SG NK cells in pSS model mice. Isotype control antibody (Ab) was used in place of anti-NKp46 Ab. Scale bar: 50 μm. **(C)** Alexa 568 (red)-conjugated anti-NKp46 Ab, and Alexa 488 (green)-conjugated anti-CD3, CD19, and CD11c Abs were used for the detection of NKp46^+^ NK cells in SG tissues of SS model mice. A photo is representative of five samples. Scale bar: 20 μm. **(C)** The number of NKp46^+^ SG NK cells at 6 and 12 weeks of age was measured using automatic cell counter and flow cytometric analysis. Data are presented as the means ± standard deviation (SD) with five or seven mice from each group. *P < 0.05 (two-way ANOVA). **(D)** The number of NKp46^+^ splenic NK cells at 6 and 12 weeks of age was measured, and data are presented as the means ± SD with five or seven mice from each group.

In this mouse model of pSS, autoimmune lesions in the SGs are observed from 6 weeks of age in females dominantly (42, 43). Lymphocytic infiltration around the salivary duct with the destruction of acinar cells was observed in pSS model mice at 8 weeks of age (Figure 1A). A report demonstrated that the number of NK cells, among ILCs, was largely increased within SG tissue (24). Immunohistochemical analysis of the SG tissues of pSS model mice revealed that SG NKp46^+^ NK cells were observed in the inflammatory lesions, whereas a few NK cells were observed in the SG tissue of control mice (Figure 1B). Flow cytometric analysis revealed that the number of SG NK cells was significantly higher in pSS model mice than in control mice at 6 weeks of age (Figure 1C). Moreover, in pSS model mice, the number of SG NK cells was significantly higher at 6 weeks of age than that at 12 weeks of age (Figure 1C). In contrast, no difference in the number of splenic NK cells were observed between pSS model and control mice at both 6 and 12 weeks of age (Figure 1D). These findings imply that an increase number of SG NK cells may be associated with the development of autoimmune lesions in pSS model mice.

### Differentiation of salivary gland natural killer cells in primary Sjögren’s syndrome model mice

**FIGURE 2:**
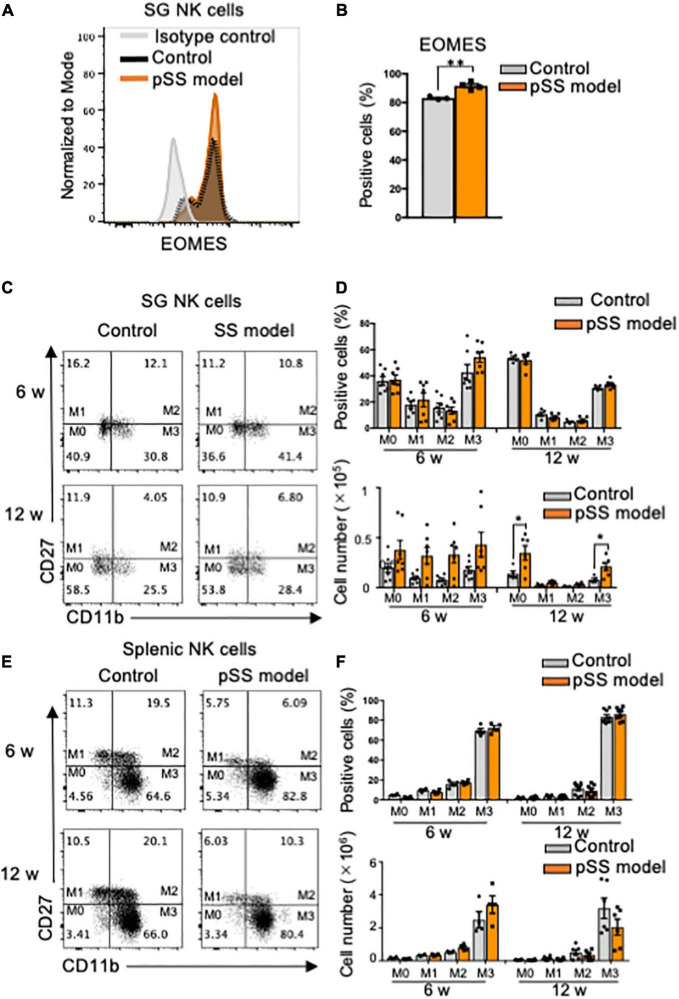
Phenotype and maturation of SG NK cells in pSS model mice. **(A)** The intracellular expression of EOMES in NKp46^+^ SG NK cells was analyzed using flow cytometry. An overlayed histogram shows EOMES expression. The results are representative of each group. **(B)** The proportion of EOMES^+^ cells among NKp46^+^ SG NK cells at 8 weeks of age was evaluated using flow cytometric analysis. Data are presented as the mean ± SD with four mice from each group. **P < 0.005 (two-tailed Student’s *t*-test), vs. control. The experiments were repeated twice and the results are representative of them. **(C)** NK cell maturation was evaluated using CD11b and CD27 expression by flow cytometric analysis. Flow cytometric panels are representative of each group at 6 and 12 weeks of age. Maturation stage is showed as M0 to M3. M0: CD11b^–^CD27^–^, M1: CD11b^–^CD27^+^, M2: CD11b^+^CD27^+^, M3: CD11b^+^CD27^–^. **(D)** The proportion (upper figure) and cell number (lower figure) of each stage in SG NK cells at 6 and 12 weeks of age were measured by flow cytometric analysis. Data are presented as the means ± SD with seven mice from each group. *P < 0.005 (two-way ANOVA), vs. control. **(E)** NK cell maturation in the spleen was evaluated using CD11b and CD27 expression by flow cytometric analysis. **(F)** The proportion (upper) and cell number of each stage in splenic NK cells at 6 and 12 weeks of age were measured by flow cytometric analysis. Data are presented as the means ± SD with seven mice from each group.

Immature and mature NK cells express Eomesodermin (EOMES), a key transcription molecule (7, 44). When the expression of EOMES in SG NK cells of pSS model mice was detected by intracellular flow cytometric analysis, a significant increase in the proportion of EOMES^+^ cells of SG NK cells in pSS model mice was observed compared with that of control mice (Figures 2A,B). To further understand the differentiation and maturation of SG NK cells in pSS model mice, the differential expression of CD27 and CD11b was examined to discriminate four consecutive stages of NK cell maturation: CD27^+^CD11b^–^ (mature stage 1: M1), CD27^+^CD11b^+^ (M2), CD27^–^CD11b^+^ (M3), and CD27^–^CD11b^–^ (immature stage: M0) NK cells (45–47). While the proportion of M0–M3 subpopulations of SG NK cells in pSS model mice was similar to that in control mice at 6 and 12 weeks of age (Figures 2C,D), the number of all the subsets in SG NK cells in pSS model mice increased, but not significantly, compared with that in control mice (Figures 2C,D). Additionally, the number of M0 and M3 SG NK cells was significantly higher in pSS model mice than in control mice at 12 weeks of age (Figure 2D). No differences were found in the proportion and number of each NK cell subset in spleen between control and pSS model mice (Figures 2E,F). The results suggest that the unique maturation of SG NK cells is promoted in pSS model mice.

### Unique phenotypes of salivary gland natural killer cells in primary Sjögren’s syndrome model mice

**FIGURE 3:**
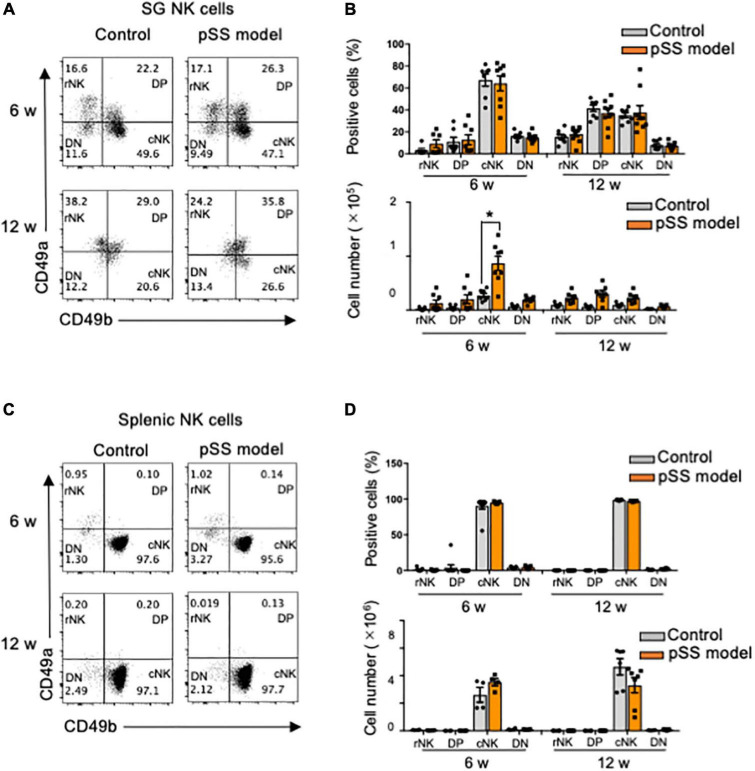
Differentiation of SG NK cells in pSS model mice. **(A)** NK cell differentiation was evaluated using CD49a and CD49b expression by flow cytometric analysis. Flow cytometric panels are representative of each group at 6 and 12 weeks of age. Each subset is showed as DN: CD49a^–^CD49b^–^, rNK: CD49a^+^CD49b^–^, DP: CD49a^+^CD49b^+^, and cNK: CD49a^–^CD49b^+^. **(B)** The proportion (upper figure) and cell number (lower figure) of each subset in SG NKp46^+^NK cells at 6 and 12 weeks of age were measured by flow cytometric analysis. Data are presented as the means ± SDs with seven mice from each group. *P < 0.005 (two-way ANOVA), vs. control. **(C)** NK cell subsets in the spleen were evaluated using CD49a and CD49b expression by flow cytometric analysis. **(D)** The proportion (upper) and cell number of each subset in splenic NKp46^+^ NK cells at 6 and 12 weeks of age were measured by flow cytometric analysis. Data are presented as the means ± SD with four to seven mice from each group.

SG ILCs express several unique phenotypes, such as CD49a and CD49b, which promote lymphocyte homing to non-lymphoid tissues (24, 48): CD49a^+^CD49b^–^ (resident NK cell: rNK), CD49a^+^CD49b^+^ (DP), CD49a^–^CD49b^+^ (conventional NK cell: cNK), and CD49a^–^CD49b^–^ (DN) (24). CD49a^+^NK cells are considered tissue-resident NK cells (24.47). No changes in the proportions of rNK, DP, cNK, and DN cells among SG NK cells were found in control and pSS model mice at 6 and 12 weeks of age (Figures 3A,B). Regarding cell number, the number of cNK cells in pSS model mice was significantly increased compared with that in control mice at 6 weeks of age whereas the number of cNK cells was drastically reduced at 12 weeks of age (Figure 3B). No difference in the number of the other NK cell subsets was observed between control and pSS model mice (Figure 3B). Almost all splenic NK cells had the cNK cell phenotype, and no differences in the proportion of splenic cNK cells were observed between control and pSS model mice at 6 and 12 weeks of age (Figures 3C,D). The phenotypic changes in SG NK cells may be occurred during development of inflammatory lesions in pSS model mice.

### Cytotoxic features of salivary gland natural killer cells in primary Sjögren’s syndrome model mice

**FIGURE 4:**
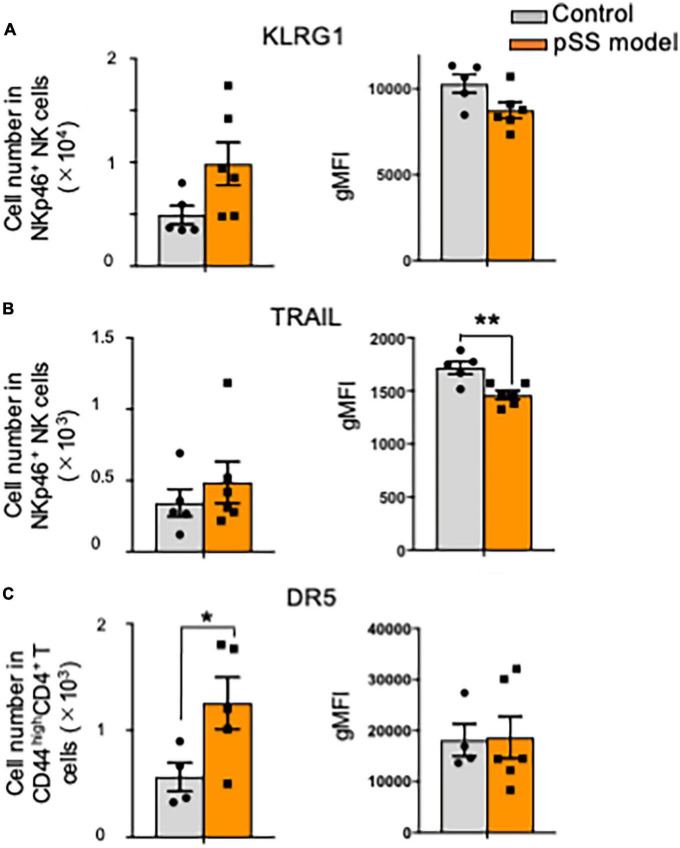
Cytotoxic signature of SG NK cells against T cells in pSS model mice. **(A)** The number (left) and geometric mean fluorescence intensity (gMFI) (right) of KLRG1^+^ SG NK cells were evaluated by flow cytometric analysis. Data are presented as the means ± SD with five or six mice from each group. **(B)** The number (left) and gMFI (right) of TRAIL^+^ SG NK cells were evaluated by flow cytometric analysis. Data are presented as the means ± SD with five or six mice from each group. **P < 0.0005 (two-tailed Student’s *t*-test), vs. control. **(C)** The number (left) and gMFI (right) of DR5 SG T cells were evaluated by flow cytometric analysis. Data are presented as the means ± SD with five or six mice from each group. *P < 0.005 (two-tailed Student’s *t*-test), vs. control.

Killer cell lectin-like receptor subfamily G member 1 (KLRG1) is a lymphocyte co-inhibitory or immune checkpoint receptor, expressed predominantly on late-differentiated effector and effector memory CD8^+^ T and NK cells (49, 50). The expression of KLRG1 is associated with the cytotoxic effector function of NK cells (51, 52). No difference in the expression of KLRG1 in SG NK cells was observed between control and pSS model mice in the terms of proportion, cell number, and geometric mean fluorescence intensity (gMFI) (Figure 4A). Moreover, tumor necrosis factor (TNF)-related apoptosis-inducing ligand (TRAIL)-expressing SG NK cells control CD4^+^ T cell response during chronic viral infection to limit autoimmunity (53). Although no changes in the proportion and number of TRAIL^+^ SG NK cells were found in control and pSS model mice at 12 weeks of age, the gMFI of TRAIL expression in SG NK cells of pSS model mice was significantly downregulated compared with that of control mice (Figure 4B). Furthermore, one of the receptors for TRAIL, death receptor 5 (DR5), on CD4^+^T cells plays an important role in T cell regulation through NK cells in the SGs during chronic viral infection (53). The number of activated DR5^+^ CD44*^high^* CD4^+^T cells in the SGs from pSS model mice was significantly higher than that in the SGs from control mice, whereas no changes in the proportion and gMFI of activated DR5^+^ CD44*^high^* CD4^+^T cells were observed between control and SS model mice (Figure 4C).

### Interferon-γ production from T cells and natural killer cells in the target organ of primary Sjögren’s syndrome model mice

**FIGURE 5:**
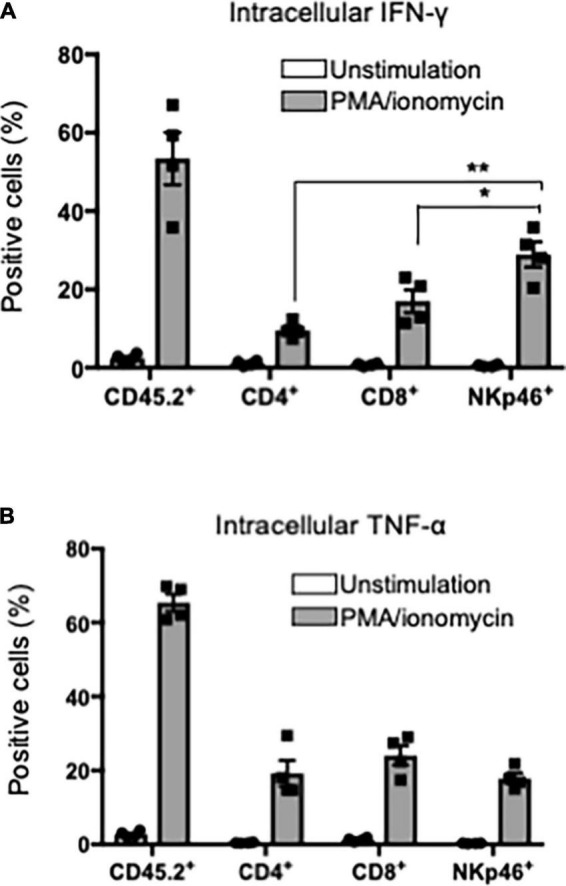
IFN-γ production in T and NK cells in SG. CD45.2^+^ cells were purified from the SG tissues of pSS model mice and the purified cells were stimulated with PMA/ionomycin for 24 h. Intracellular IFN-γ or TNF-α expression was evaluated by flow cytometric analysis. **(A)** Intracellular IFN-γ expression in CD45.2^+^, CD4^+^, CD8^+^ T cells, and NKp46^+^NK cells was analyzed. The proportions of IFN-γ^+^ cells were evaluated, and data are presented as the means ± SD with four mice from each group. *P < 0.005, **P < 0.0005 (two-tailed Student’s *t*-test). **(B)** Intracellular TNF-α expression in CD45.2^+^, CD4^+^, CD8^+^ T cells, and NKp46^+^NK cells was analyzed, and the proportion of TNF-α^+^ cells were evaluated, and data are presented as the means ± SD with four mice from each group. The experiments were repeated three times and the results are representative of them.

Th1 cells that produce inflammatory cytokines, such as IFN-γ, play a key role in the pathogenesis of pSS (35, 38). Particularly, IFN-γ from SG tissues in patients with pSS and pSS model mice is associated with the activation of T cells, antigen-presenting cells, and epithelial cells (39). When CD45.2^+^ lymphoid cells within SG tissues, including T cells and NK cells, in pSS model mice were stimulated with phorbol 12-myristate 13-acetate (PMA) for 24 h, IFN-γ production could be detected by flow cytometric analysis with intracellular staining. Surprisingly, the production level of IFN-γ in SG NK cells was significantly higher than that in CD4^+^ and CD8^+^ T cells within SG tissues (Figure 5A). In contrast, no difference in the intracellular TNF-α production was found among CD4^+^, CD8^+^ T cells, and NK cells in SG tissues in pSS model mice (Figure 5B). These results indicate that SG NK cells can produce abundant IFN-γ in the target organ of pSS.

### Therapeutic effects of natural killer cell depletion on autoimmune lesions in primary Sjögren’s syndrome model mice

**FIGURE 6:**
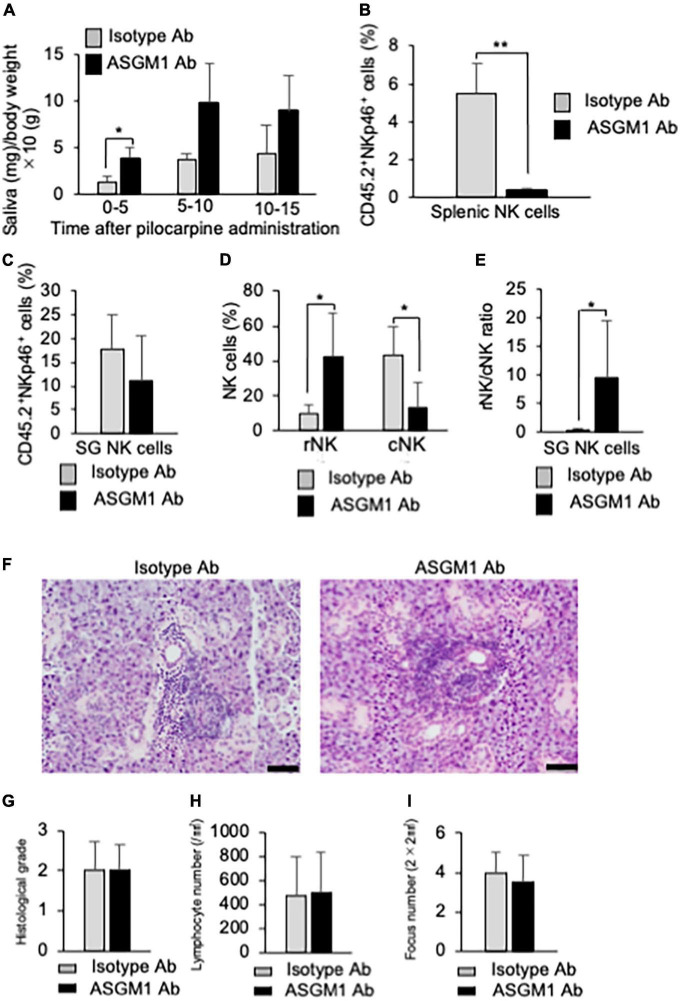
Effects of NK cell depletion on the SGs in pSS model mice. **(A)** The saliva weight of isotype antibody (Ab) and anti-asialo-GM1 (ASGM1) Ab-treated pSS model mice were measured from 5 min until 15 min after the oral administration of pilocarpine. Data are presented as the means ± SD of saliva weight (mg)/body weight × 10 (g) with four mice from each group. *P < 0.05 (two-way ANOVA). **(B)** The depletion of NK cells in the spleen was confirmed by flow cytometric analysis. Data are presented as the means ± SD of CD45.2^+^NKp46^+^ NK cells (%) with four mice from each group. **P < 0.005 (two-tailed Student’s *t*-test). **(C)** The proportion of SG NK cells was evaluated by flow cytometric analysis. Data are presented as the means ± SD of CD45.2^+^NKp46^+^ NK cells (%) with four mice from each group. **(D)** Mature rNK (CD49a^+^CD49b^–^) and cNK (CD49a^–^CD49b^+^) cells among CD45.2^+^NKp46^+^ NK cells were measured by flow cytometric analysis. Data are presented as the means ± SD with four mice from each group. *P < 0.05 (two-tailed Student’s *t*-test). **(E)** The ratio of rNK to cNK cells was presented as the means ± SD with four mice from each group. *P < 0.05 (two-tailed Student’s *t*-test). **(F)** Pathological analysis of SG tissues in isotype Ab or ASGM1 Ab-treated pSS model mice. Sections stained with HE were used. Photos of are representative of each group. Scale bar: 50 μm. **(G)** Histological grade of SG tissues was evaluated using HE-stained sections. Data are presented as the means ± SD with four mice from each group. **(H)** The lymphocyte number of the SG/mm^2^ was measured using histological sections. Data are presented as the means ± SD with four mice from each group. **(I)** Focus number/2 × 2 mm^2^ was measured using histological sections. Data are presented as the means ± SD with four mice from each group. The experiments were repeated three times and the results are representative of them.

To confirm the effects of NK cell depletion on inflammatory lesions in the SGs in pSS model mice, anti-asialo-GM1 antibody (ASGM1 Ab) was intraperitoneally injected into mice twice a week from 9 to 12 weeks of age. To examine the functions of the SG, saliva weight was measured after intraoral administration of pilocarpine, which is an inducer of saliva secretion as a muscarine receptor antagonist. Five minutes after pilocarpine injection, the saliva weight of ASGM1 Ab-treated pSS model mice significantly increased compared with that of control mice (Figure 6A). After 5 min, the saliva weight in ASGM1 Ab-treated mice increased in contrast to that in control mice; however, the difference was not statistically significant (Figure 6A). We have repeated the experiments three times, and obtained the similar results that saliva secretion of ASGM1 Ab-treated mice was significantly higher than control mice only at the early stage after stimulation. A significant difference in terms of the depletion of NKp46^+^NK cells in CD45.2^+^ cells in the spleen was observed between control and ASGM1 Ab-treated mice (Figure 6B). However, the proportion of NKp46^+^ SG NK cells among CD45.2^+^ cells in ASGM1 Ab-treated mice did not significantly decrease (Figure 6C). The proportion of CD49a^+^CD49b^–^ rNK cells among SG NK cells was significantly higher in ASGM1 Ab-treated mice than in control mice, whereas the proportion of CD49a^–^CD49b^+^ cNK cells was significantly decreased following ASGM1 Ab administration (Figure 6D). The ratio of rNK/cNK cells in the SG was significantly higher following ASGM1 Ab administration (Figure 6E). To understand the pathological mechanism of the effectiveness of NK cell depletion, SG tissues from pSS model mice were histopathologicaliy analyzed. Inflammatory lesions, including lymphocyte infiltration around ductal cells, were found in both isotype control and ASGM1 Ab treated mice (Figure 6F). Three evaluations for inflammatory severity were analyzed by histological grading, lymphocyte number/mm^2^, and fucus number/2 × 2 mm^2^. No differences in inflammatory severity were observed between isotype control and ASGM1 Ab-treated mice (Figures 6G,H,I). In this experiment, ASGM1 Ab treatment could recover the salivation, but not inflammatory lesions in pSS model mice.

## Discussion

Natural killer cells contribute to various immune responses. In this study, NK cells in the target organ of pSS were focused on, and the association between NK cells and the formation of autoimmune lesions was analyzed using pSS model mice. The maturation of SG NK cells in pSS model mice was promoted in response to the formation of autoimmune lesions in SG tissues. Additionally, mature rNK cells may control the recruitment of cNK cells to maintain homeostasis of the immune environment in SG tissues. Although the depletion of NK cells caused by the administration of ASGM1 Ab failed to protect lymphocytic infiltration in the SGs of pSS model mice, saliva secretion increased in the Ab-treated mice. These results suggest that SG NK cells may contribute to the cytotoxic activity of T cells during the development of pSS.

NKp46^+^ SG NK cells were detectable in the marginal region of the foci of autoimmune lesions in pSS model mice. This finding was similar to that of lip biopsy materials in patients with SS (54). The main populations in the inflammatory lesion of SGs from the pSS model mice as well as the patients with SS are T and B cells (42). SG NK cells may support the autoimmune response by T or B cells. In this model, inflammatory lesions are observed from 6 week of age (42, 43). A significantly increased number of SG NK cells, including several cNK cells, were observed at 6 weeks of age in pSS model mice, whereas no difference was observed at 12 weeks of age, suggesting that increased SG NK cells may play a pathogenic role in the development of pSS. In addition, our results suggest that the phenotypic changes in SG NK cells may contribute to the development of autoimmune lesions in pSS model mice. A recent report as for the other SS model mice demonstrates that the proportion of NK1.1^+^ SG NK cells was significantly higher in C57BL/6 mice injected with an antagonist of the stimulator of IFN gene (STING) protein than in control mice (55), resembling the increase of SG NK cells during the onset in our model mice. Moreover, it is reported that the number of SG NK cells of normal mice is increased with aging (24). In our study, SG NK cells between 6 and 12 weeks of age were compared focusing on the development of inflammatory lesions in pSS model mice. More aged mice should be analyzed to understand the relationship between aging and NK cells in autoimmune lesions.

The maturation of SG NK cells was promoted at 6 weeks of age in pSS model mice. This result suggests that SG NK cells are accompanied by T cell response in the development of autoimmune lesions in pSS. The intracellular expression of EOMES in SG NK cells was higher in pSS model mice than in SG NK cells in control mice. EOMES is a key transcription regulator of mature NK cell homeostasis and cytotoxicity (56). As for cytotoxicity against activated T cells by SG NK cells in pSS model mice, KLRG1 or TRAIL expression was not upregulated, suggesting that the cytotoxic activity of SG NK cells is inadequate against autoreactive and activated T cells in the target organ of pSS. A report demonstrated that TRAIL^+^ NK cells control CD4^+^ T cell responses in the SGs during chronic viral infection to limit autoimmunity (53). In our model, TRAIL^+^ NK cells failed to control CD4^+^ T cell response attacking to target cells, unlike the condition during chronic viral infection. Our results suggest that SG NK cells fail to regulate activated or autoreactive CD4^+^ T cells *via* TRAIL/DR5 in the target organ of pSS model mice.

Interferon-γ plays potent roles in the onset or development of pSS (33, 38, 39). The source of IFN-γ in SG tissue contains various cells, such as CD4^+^ T cells, CD8^+^ T cells, macrophages, NK cells, and ductal epithelial cells (38–40). In this study using *in vitro* stimulation with PMA/ionomycin, SG NK cells produced higher levels of IFN-γ than CD4^+^ and CD8^+^ T cells among CD45.2^+^ hematopoietic cells in the SGs of pSS model mice. During the development process of SS, IFN-γ contributes to the activation of Th1 cells, the upregulation of MHC Class II of antigen-presenting or epithelial cells, the production of IFN-γ-inducible proteins, and the activation of various immune cells expressing IFN-γ receptor (37). A significantly increased number of cNK cells in the SGs of pSS model mice were observed at 6 weeks of age. Autoimmune lesions were found from 6 weeks pf age in this model. Plenty of IFN-γ produced from SG NK cells together with T cells can promote the autoimmune response within the target organ to form inflammatory and destructive lesions in pSS model mice.

The therapeutic effect of ASGM1 Ab injection was found on saliva secretion in pSS model mice. In contrast, the infiltration of lymphocytes into the SGs was not altered by the administration of ASGM1 Ab. The autoimmune response to the destruction of SG cells in pSS is considered to be dependent on T cell cytotoxicity (57). The findings in this study indicate that cNK cells in the SGs may promote T cell cytotoxicity. NK cells control virus-infected T cells to inhibit viral inflammation (25, 58, 59). Moreover, no changes in the proportion of SG NK cells were observed in ASGM1 Ab-treated pSS model mice, whereas the ratio of rNK to cNK cells in the SG was greatly increased. These findings suggest that the homeostasis of total SG NK cells is maintained by rNK cells.

We determined the dose of *in vivo* ASGM1 Ab injection by which systemic NK cells were completely deleted. In this study, the almost depletion of NK cells was confirmed in the spleen of ASGM1 Ab-treated pSS model mice. However, although cNK cells were depleted in the SG of ASGM1 Ab-treated pSS model mice, the proportion of rNK cells was conversely increased. ASGM1 Ab may stimulate rNK cells rather than deplete them. As the recovery of saliva secretion was confirmed by ASGM1 Ab administration, the therapeutic administration of ASGM1 Ab was effective in recovering SG function.

The differentiation, phenotype, and function of NK cells are so complex that various cell markers are used to distinguish differential subsets of NK cells (47). In this study, various NK cell markers were examined to detect SG NK cells in control and pSS model mice. As for NK1.1, one of the most common markers of NK cell, the expression of NK1.1 in SG NK cells was dull, and therefore, NKp46 was used throughout all the experiments. The population that expressed NKp46 was used to discriminate various subsets of NK cells in the SG. Additionally, the distribution of NKp46^+^ NK cells in the inflammatory lesions of patients with pSS is similar to that observed in pSS model mice (54).

## Conclusion

In summary, SG NK cells play a pathogenic role in the development of autoimmune lesions in pSS model mice by prominently upregulating of IFN-γ within the target organ. In addition, controlling the proportion of rNK and cNK cells in SG tissues influences T cell cytotoxicity or autoreactivity in pSS model mice, indicating that rNK cells may play a potent role in regulating autoreactive T cells in the target organ of pSS. These results can be used develop new therapeutic strategies targeting NK cells for autoimmune diseases, including pSS.

## Data availability statement

The raw data supporting the conclusions of this article will be made available by the authors, without undue reservation.

## Ethics statement

This animal study was reviewed and approved by Committee on Animal Experiments of Tokushima University and Biological Safety Research Center, Japan (Permit No: T-2021-48).

## Author contributions

NI supervised the study, acquired the funding, and wrote the original draft. MS and RA conceptualized, analyzed data, investigated, and wrote the original draft. HTaw, RN, HTan, KT, YK, KO, AU, and TT contributed to the analyzed data and investigated. All authors contributed to the article and approved the submitted version.
